# Supplementary material for: Heparin-based hydrogel scaffolding alters the transcriptomic profile and increases the chemoresistance of MDA-MB-231 triple-negative breast cancer cells
Source: Biomater Sci. 2020 Feb 13;8(10):2786–96. doi: 10.1039/c9bm01481k (PMC7497406; doi:10.1039/c9bm01481k)
Supplement: Supplementary file 2 [file BM-008-C9BM01481K-s002.zip › Supplementary File 4/HP-B hydrogel + EGF (s)vControl/Pathways/my_analysis.Gsea.1545201170987/HALLMARK_HEDGEHOG_SIGNALING.html]

Details for gene set HALLMARK\_HEDGEHOG\_SIGNALING[GSEA]

|  || Dataset | expr.class.cls#HYDEGF\_versus\_CONTROL.class.cls#HYDEGF\_versus\_CONTROL\_repos |
| Phenotype | class.cls#HYDEGF\_versus\_CONTROL\_repos |
| Upregulated in class | CONTROL |
| GeneSet | HALLMARK\_HEDGEHOG\_SIGNALING |
| Enrichment Score (ES) | -0.29606768 |
| Normalized Enrichment Score (NES) | -0.96518636 |
| Nominal p-value | 0.48642534 |
| FDR q-value | 0.573862 |
| FWER p-Value | 1.0 |
Table: GSEA Results Summary

  

Fig 1: Enrichment plot: HALLMARK\_HEDGEHOG\_SIGNALING      
 Profile of the Running ES Score & Positions of GeneSet Members on the Rank Ordered List

  

| PROBE | DESCRIPTION (from dataset) | GENE SYMBOL | GENE\_TITLE | RANK IN GENE LIST | RANK METRIC SCORE | RUNNING ES | CORE ENRICHMENT || 1 | CRMP1 | na |  |  | 829 | 1.802 | 0.0642 | No |
| 2 | TLE1 | na |  |  | 2842 | 1.043 | 0.0214 | No |
| 3 | RASA1 | na |  |  | 4413 | 0.747 | -0.0159 | No |
| 4 | RTN1 | na |  |  | 5088 | 0.640 | -0.0129 | No |
| 5 | NF1 | na |  |  | 5105 | 0.636 | 0.0242 | No |
| 6 | PLG | na |  |  | 6144 | 0.483 | -0.0011 | No |
| 7 | OPHN1 | na |  |  | 6170 | 0.480 | 0.0262 | No |
| 8 | CELSR1 | na |  |  | 6956 | 0.378 | 0.0077 | No |
| 9 | CDK5R1 | na |  |  | 7158 | 0.350 | 0.0181 | No |
| 10 | TLE3 | na |  |  | 7257 | 0.336 | 0.0330 | No |
| 11 | CDK6 | na |  |  | 7387 | 0.319 | 0.0453 | No |
| 12 | ETS2 | na |  |  | 7527 | 0.301 | 0.0560 | No |
| 13 | PML | na |  |  | 7928 | 0.252 | 0.0502 | No |
| 14 | NRP2 | na |  |  | 8075 | 0.232 | 0.0564 | No |
| 15 | UNC5C | na |  |  | 8378 | 0.199 | 0.0525 | No |
| 16 | PTCH1 | na |  |  | 9867 | 0.027 | -0.0235 | No |
| 17 | L1CAM | na |  |  | 10803 | -0.078 | -0.0676 | No |
| 18 | NKX6-1 | na |  |  | 10893 | -0.092 | -0.0667 | No |
| 19 | LDB1 | na |  |  | 11744 | -0.195 | -0.0995 | No |
| 20 | NRCAM | na |  |  | 13281 | -0.383 | -0.1567 | No |
| 21 | ACHE | na |  |  | 13812 | -0.461 | -0.1569 | No |
| 22 | DPYSL2 | na |  |  | 15353 | -0.697 | -0.1957 | No |
| 23 | NRP1 | na |  |  | 17279 | -1.174 | -0.2261 | Yes |
| 24 | VLDLR | na |  |  | 17779 | -1.371 | -0.1704 | Yes |
| 25 | SHH | na |  |  | 18488 | -1.830 | -0.0982 | Yes |
| 26 | SCG2 | na |  |  | 18839 | -2.267 | 0.0187 | Yes |
Table: GSEA details [plain text format]

  

Fig 2: HALLMARK\_HEDGEHOG\_SIGNALING      
 Blue-Pink O' Gram in the Space of the Analyzed GeneSet

  

Fig 3: HALLMARK\_HEDGEHOG\_SIGNALING: Random ES distribution      
 Gene set null distribution of ES for **HALLMARK\_HEDGEHOG\_SIGNALING**
